# Equine neutrophils selectively release neutrophil extracellular traps in response to chemical and bacterial agonists

**DOI:** 10.3389/fvets.2025.1512343

**Published:** 2025-02-24

**Authors:** Breanna J. Sheahan, Alicia G. Schubert, William Schubert, M. Katie Sheats, Lauren V. Schnabel, Jessica M. Gilbertie

**Affiliations:** ^1^Department of Clinical Sciences, College of Veterinary Medicine, North Carolina State University, Raleigh, NC, United States; ^2^Comparative Medicine Institute, North Carolina State University, Raleigh, NC, United States; ^3^College of William & Mary, Williamsburg, VA, United States; ^4^Department of Biomedical Sciences and Pathobiology, Virginia-Maryland College of Veterinary Medicine, Blacksburg, VA, United States

**Keywords:** equine, neutrophils, neutrophil extracellular traps, citrullinated histones, NADPH-oxidase pathway, autophagy

## Abstract

**Introduction:**

Neutrophil extracellular traps (NETs) play a significant role in response to a variety of infectious and inflammatory stimuli in human and veterinary medicine. Although entrapment of bacteria can be an important function of NETs, the exuberant release of DNA and other intracellular molecules has also been negatively implicated in the pathogenesis of different diseases. Thus, NET formation must be tightly controlled and represents an opportunity for therapeutic interventions. Horses are particularly sensitive to bacterial stimuli that have previously been shown to cause NETs in other species, but the species-specific processes that control NET release have not been fully elucidated.

**Methods:**

The purpose of this study was to compare the magnitude of response of equine neutrophils to different chemical and bacterial stimuli, including phorbol 12-myristate 13-acetate (PMA), a calcium ionophore (A23187), *Staphylococcus aureus*, and *Escherichia coli*. In addition, we investigated whether ex vivo equine NET formation is controlled by the NADPH-oxidase (NOX) pathway and by autophagy, both of which control NET formation in other species.

**Results:**

We demonstrated that equine neutrophils produce robust NETs in response to calcium ionophore and *E. coli* stimuli and produce fewer NETs in response to PMA and *S. aureus*. Both NOX-dependent and NOX-independent pathways of NET formation were identified in equine neutrophils. Autophagy inhibition altered the mechanics of NET release, by reducing the amount of extracellular DNA stranding.

**Discussion:**

These results provide insight into equine-specific neutrophil biology, which could be key for managing equine diseases such as asthma and laminitis.

## 1 Introduction

As part of the innate immune system, neutrophils play a critical role in pathogen control and clearance as well as immune cell recruitment. Neutrophils extrude neutrophil extracellular traps (NETs) (1–3), in addition to their traditionally described functions of phagocytosis and respiratory burst (4). NETs consist of extracellular DNA that has been actively extruded from the nucleus of the neutrophil and is decorated with antimicrobial proteins such as myeloperoxidase. Deposition of citrulline onto histones is a hallmark of active NET formation, distinguishing this cellular process from cellular death that results in extracellular DNA (5, 6). NETs control pathogens by physically immobilizing and chemically impairing them. However, NET formation has been demonstrated to have negative effects on the host (7, 8), including microthrombi (9, 10), pro-inflammatory stimulation, self-antigen sensitization (11), and off-target tissue destruction (12). Thus, the process of NET formation should be tightly controlled in response to the instigating stimulus.

The pathways controlling NET formation are dependent on the stimulus presented to the neutrophils (13). In response to stimulation with chemical agonist phorbol 12-myristate 13-acetate (PMA), NET formation is dependent on NADPH-oxidase (NOX) activation of protein kinase C, also referred to as “suicidal NETosis” (14, 15). PMA has been used as a reference for ex vivo NET experiments in multiple species, including human (2, 10, 16), murine (17), canine (18), and equine (19) neutrophils. NOX-dependent NET formation occurs in response to certain bacterial and fungal stimuli, including *Streptococcus agalactiae* and *Candida albicans* (20). In human ex vivo studies, NET formation occurs within 2–4 h of stimulation with PMA and is concentration dependent until maximal stimulation is reached (16, 21). In contrast, a rapid (5–90 min) NOX-independent NET release from human neutrophils occurs in response to calcium ionophores such as A23187, also known as “vital NETosis” (13, 16, 21). The NOX-independent pathway controls NET release from human neutrophils in response to some species of bacteria, including *Staphylococcus aureus, Salmonella typhimurium*, and *Shigella flexneri* (1, 13).

Autophagy is a ubiquitous cellular process to recycle cellular components which controls NET formation in human neutrophils exposed to PMA (22) and lipopolysaccharide (LPS) (23). Autophagy has also been demonstrated in human and mouse studies to be important for neutrophil-mediated inflammation (24, 25), phagocytosis (26), and neutrophil differentiation (27). Inhibition of autophagy represents a potential therapeutic target for minimizing the destructive effects of NET formation (23).

In horses, NETs have been implicated in the pathogenesis of equine asthma, laminitis, equine recurrent uveitis, and endometritis (19, 28–31). Plasma cell-free DNA (cfDNA), generated by cellular apoptosis, necrosis, and NET formation, is increased in a subset of colic patients, although it is unknown if this correlates to increased circulating NETs (32). Studies on ex vivo NET formation in equine neutrophils demonstrated that these neutrophils release NETs in response to PMA, A23187, and Platelet-Activating Factor (33, 34). No NET formation was observed in LPS (*Escherichia coli* O111:B4)-stimulated equine neutrophils, although no bacterial stimulation was used in this study (33). Horses are uniquely sensitive to circulating bacterial components, including LPS from gram negative bacteria such as *E. coli*. In addition, horses undergo massive and rapid neutrophil margination and extravasation in response to inflammatory stimuli (35). Given these unique species characteristics, we hypothesize that the horse may exhibit exuberant NET formation in the face of commonly-studied stimuli such as bacteria. Limiting NET formation may represent a potential therapeutic target in horses to reduce side-effects from excessive NET release.

The objective of this study was to characterize NET formation in equine peripheral blood neutrophils isolated from healthy horses in response to a variety of stimuli. In addition, impairment of NET formation was studied via NAPDH-oxidase inhibition and autophagy inhibition. We identified that equine neutrophils produce robust NETs in response to calcium ionophore and *E. coli*, but not PMA or *S. aureus*. In addition, we demonstrated that NET extrusion induced by calcium ionophore can be limited by autophagy inhibition.

## 2 Materials and methods

### 2.1 Ethics statement

The use of animals for this study was reviewed and approved by NC State University’s IACUC (protocol # 19-628).

### 2.2 Neutrophil isolation

Whole blood was collected via jugular venipuncture from healthy thoroughbred or thoroughbred cross horses (*n* = 8, 4 mares, 4 geldings) in our closed research herd used only for blood and bone marrow collection. These horses ranged from 8 to 20 years old with a median age of 12 years old. No previous or ongoing medical concerns were present. These horses had normal physical examinations prior to every donation and had complete blood counts obtained four times a year. On these complete blood counts, the horses were only used for blood collection if their results were within reference range for neutrophil concentration and percentage of total leukocytes. The blood was collected into a 60 mL syringe containing 6 mL of acid citrate dextrose (ACD), each for a total volume of 60 mL per horse. Erythrocytes were allowed to settle for 30 min in the syringe and the layer above the erythrocytes containing the leukocytes, platelets, and plasma (approximately 30 mL) termed L-PRP was then transferred to a 50 mL conical tube (36, 37). L-PRP was carefully layered v/v over Ficoll Paque Plus (GE Healthcare-Life Sciences, Marlborough, MA) and centrifuged at 695 g for 15 min without brake. The cell pellet containing the remaining erythrocytes and leukocytes was resuspended in 1 mL of 1X Dulbecco’s phosphate-buffered saline (DPBS). Hypotonic lysis of the remaining erythrocytes was performed by adding 9 mL of sterile deionized water and inverting slowly 10 times before adding 1 mL of 10X DPBS to restore an isotonic solution. Neutrophils were pelleted via centrifugation at 400 g for 5 min and resuspended in 10 mL RPMI 1640 with 10% Fetal Bovine Serum (FBS) and counted using a Cellometer^®^ Auto 2000 and ViaStain™ AOPI Staining Solution (Nexcelom Bioscience LLC, Lawrence, MA, United States). The final neutrophil concentration was then adjusted to 1 × 10^6^/mL. This method has demonstrated consistent recovery of >96% neutrophils as a final percentage of leukocytes (37). Neutrophils were confirmed to be at least 97% viable prior to use for experiments. Neutrophils were allowed to rest for 1 h at 37°C, 5% CO_2_, and 90% humidity prior to being stimulated with chemical agonists or challenged with bacteria.

The final total number of cells available for ex vivo testing was a mean of 1.69 × 10^8^ ± 0.2 × 10^8^ per sample. Based on the historical complete blood cell count data from each horse, the estimated mean recovery rate of neutrophils per sample (54 mL whole blood) was 69.2% (range 52.5–79%). All assays on each set of isolated neutrophils from an individual horse were performed in triplicate. For statistical analysis, normality was assessed with Shapiro–Wilk test, followed by parametric analysis. A two-way repeated measures ANOVA followed by Bonferroni’s *post hoc* test was performed for all measurements completed over time after neutrophil stimulation, infection, or inhibition. For area under the curve comparisons, a one-way or two-way ANOVA followed by Bonferroni’s post hoc test was performed as appropriate.

### 2.3 Neutrophil stimulation or infection

For chemical stimulation, neutrophils were added to black 96-well plates at 1 × 10^5^ in RPMI 1640 with 10% FBS (non-heat inactivated), unless otherwise indicated, at 37°C 5% CO_2_, and 90% humidity. Neutrophils were stimulated with 20, 50, 100, 200 μM PMA or 1, 2, 5, 10 μM A23187 (Cayman chemical, Ann Arbor, MI; dissolved in dimethyl sulfoxide (DMSO)) and measurements of respiratory burst or NET formation were recorded at indicated timepoints. Concentrations of PMA and A23187 were chosen based on previous literature in horses (34, 38) and humans (16), where 100 μM PMA and 5 μM A23187 are commonly cited for neutrophil stimulation. Control neutrophils had an equivalent concentration of DMSO applied. All horses demonstrated similar responses to chemical stimuli but the magnitude of the response varied between individuals. Therefore, due to high individual variation, all neutrophil responses were standardized to each animal’s response to 5 μM A23187 (100%).

For bacterial stimulation, neutrophils were added to 24-well plates at 5 × 10^5^ in RPMI 1640 with 10% FBS (non-heat inactivated), unless otherwise indicated, at 37°C, 5% CO_2_, and 90% humidity. Neutrophils were stimulated at a multiplicity of infection (MOI) of 1:1 with *S. aureus* USA300 or *E. coli* ATCC 25922 and measurements of respiratory burst or NET formation were recorded at indicated timepoints. All horses demonstrated similar responses to bacterial stimuli but the magnitude of the response varied between individuals. Due to high individual variation, all neutrophil responses were standardized to each animal’s response to *E. coli* (100%).

### 2.4 Neutrophil respiratory burst inhibition or autophagy modulation

Respiratory burst activity was inhibited by incubating neutrophils with indicated concentrations of diphenyleneiodonium chloride (DPI) (2.5–25 μM) for 30 min in RPMI 1640 with 10% FBS at 37°C and 5% CO_2._ After incubation with the inhibitor, neutrophils were spun down at 400xg for 5 min then resuspended in RPMI 1640 with 10% FBS and immediately used for experiments.

Autophagy inhibition was performed by incubating neutrophils with 25 μM hydrochloroquine (HQ) at the same time as 5 μM A23187 or an equivalent concentration of DMSO. Autophagy stimulation was performed by incubating neutrophils with 100 nM temsirolimus (TEM) at the same time as 5 μM A23187 or an equivalent concentration of DMSO. Measurements of respiratory burst or NET formation were recorded at indicated timepoints. Due to high individual variation, all neutrophil responses were standardized to each animal’s response to 5 μM A23187 (100%).

### 2.5 Respiratory burst and NET quantification

Isolated neutrophils were incubated in the presence of PMA, A23187, *S. aureus*, or *E. coli* in RPMI 1640 with 10% FBS. At indicated timepoints, 10 μM dihydrorhodamine 123 (DHR) (ThermoFisher Scientific Waltham, MA) was added to each well and incubated for 30 min prior to measuring fluorescence (excitation 485 nm, emission 528 nm) on a microtiter plate reader (Synergy™ 2, BioTek Instruments Inc., Winooski, VT) as a measurement of respiratory burst. At indicated timepoints, extracellular DNA, as a representation of NET formation was measured using cell impermeable DNA binding dye (SYTOX^®^ Green Nucleic Acid Stain, ThermoFisher Scientific Waltham, MA). 5 μM of SYTOX^®^ was added and incubated for 10 min before measuring fluorescence (excitation 485 nm, emission 528 nm).

### 2.6 Neutrophil immunofluorescence

Neutrophils were stimulated or infected as described above and incubated at 37°C, 5% CO_2_, and 90% humidity. After 4 h of incubation, plates were centrifuged at 500 g for 5 min. Each well was fixed with 4% paraformaldehyde for 15 min and permeabilized with 0.5% Triton-X 100 for 15 min. Cells were then blocked with 5% bovine serum albumin (BSA) in DPBS at room temperature (RT) for 1 h.

Immunofluorescent labeling for citrullinated Histone 3 (CitH3) was performed with rabbit anti-Histone H3 (citrulline R2 + R8 + R17) (1:250; Abcam Cat# ab5103, RRID:AB_304752) at 4°C overnight followed by goat anti-rabbit IgG (H + L) cross-adsorbed AF555 (1:500; Thermo Fisher Scientific Cat# A-21428, RRID:AB_2535849) at RT for 1 h.

Immunofluorescent labeling for myeloperoxidase in addition to CitH3 was performed by following CitH3 primary antibody application as above with goat anti-rabbit IgG (H + L) F(ab) fragment antibody (40 ug/mL; Sigma-Aldrich Cat# SAB3700970, RRID:AB_3492090) at RT for 2 h, followed by donkey anti-goat AF647 (1:500; Thermo Fisher Scientific Cat# A-21447, RRID:AB_2535864) at RT for 1 h. Following this, polyclonal rabbit anti-human myeloperoxidase (1:200; Agilent Cat# A0398, RRID:AB_2335676) was applied to the wells at 4°C overnight, followed by bovine anti-rabbit FITC (1:500; Santa Cruz Biotechnology Cat# sc-2365, RRID:AB_634836) at RT for 1 h.

Immunofluorescent labeling for autophagy protein LC3B was performed with polyclonal rabbit anti-LC3B (1:200; Sigma-Aldrich Cat# L7543, RRID:AB_796155) followed by goat anti-rabbit IgG AF555 (1:500; Thermo Fisher Scientific Cat# A-21428, RRID:AB_2535849).

Immunofluorescence control wells were incubated with rabbit IgG at the same concentration as the primary antibody (Millipore Cat# 12-370, RRID:AB_145841) followed by the appropriate secondary antibody. Nuclei were counter-stained with 4′,6-diamidino-2-phenylindole (DAPI) (BD Biosciences Cat# 564907, RRID:AB_2869624). All antibody incubations were followed by 3X washes in PBS prior to application of the next antibody or DAPI. Images were collected on an Olympus IX73 inverted scope with a DP80 camera using appropriate fluorescent channels (Olympus Corporation, Shinjuku, Tokyo, Japan).

### 2.7 Neutrophil scanning electron microscopy

After 4 h of stimulation (PMA or A23187), cells were fixed with 4% paraformaldehyde, serially ethanol dehydrated, critical point dried, sputter coated, and imaged using a Hitachi S-4700 FE-SEM with EDX or Phenom ProX Tabletop SEM/EDS at William & Mary’s Microscopy Facility.

For immunogold labeling, cells were fixed with 4% paraformaldehyde for 15 min and permeabilized with 0.5% Triton-X 100 for 15 min, then washed with 50 mM glycine in 1X DPBS. Cells were then blocked with 1% BSA in DPBS overnight at 4°C. For immunogold labeling of CitH3, cells were incubated with rabbit anti-Histone H3 (citrulline R2 + R8 + R17) (1:250; Abcam Cat# ab5103, RRID:AB_304752) at 4°C overnight, followed by goat anti-rabbit IgG H + L (10 nm gold) pre-adsorbed in 1% BSA (1:50, Abcam Cat# ab27234, RRID:AB_954427) at RT for 1 h. Cells were then incubated with 1% glutaraldehyde in DPBS for 10 min, then washed 2X with distilled water. For silver enhancement of the gold-labeling, cells were then incubated with a silver (Ag) enhancer kit (Abcam Cat# ab170732). To stop the enhancement, at 4 min post-incubation, cells were washed 2X with distilled water. Cells were then processed for SEM as above, starting with serial ethanol dehydration.

## 3 Results

### 3.1 Equine neutrophils robustly produce NETs in an NOX-independent fashion in response to calcium ionophore stimulation

**Figure 1:**
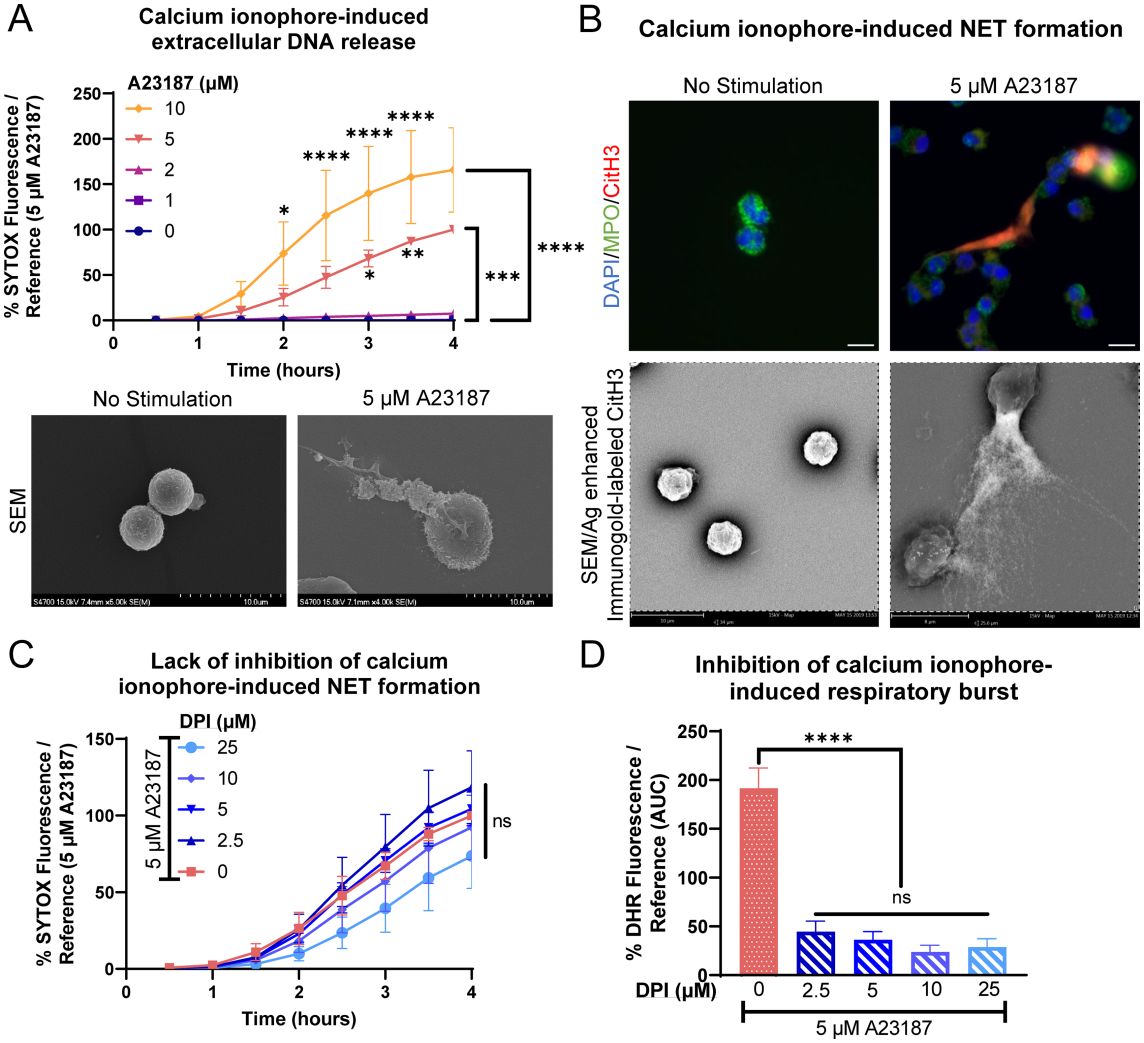
Equine neutrophils exhibit NOX-independent NET formation in response to calcium ionophore stimulation. **(A)** Measurement of extracellular DNA released over time (0–4 h) from equine neutrophils stimulated *in vitro* with increasing concentrations of calcium ionophore A23187 (1–10 μM). Extracellular DNA was measured with SYTOX fluorescence. Due to high individual variation, all neutrophil responses were standardized to each animal’s response to 5 μM A23187. *n* = 3 horses. Significance for the indicated concentrations of A23187 at the indicated timepoints: *p < 0.05; ** *p* < 0.01; *** *p* < 0.001; **** *p* < 0.0001. SEM images demonstrate extracellular contents extruded from neutrophils stimulated with 5 μM A23187. Scale bar as indicated. **(B)** Confirmation of NET formation induced 4 h post-A23187 stimulation in equine neutrophils. Myeloperoxidase (MPO) in green marks neutrophils and citrullinated H3 (CitH3) in purple identifies citrullinated histones, a marker of NET formation. Nuclei were stained with DAPI. Scale bar 10 μm. SEM images demonstrate silver-enhanced immunogold labeled citrullinated H3 (CitH3) associated with the extruded contents. Scale bar as indicated. **(C)** Measurement of NET formation over time (0–4 h) from equine neutrophils stimulated with A23187 and incubated with increasing concentrations of NADPH-oxidase inhibitor DPI. Neutrophils were incubated with DPI prior to exposure to A23187. *n* = 3 horses. ns = no significance. **(D)** Area under the curve (0–4 h) of respiratory burst from equine neutrophils stimulated with A23187 and incubated with increasing concentrations of DPI. Neutrophils were incubated with DPI prior to exposure to A23187. *n* = 3 horses. **** *p* < 0.0001.

In response to calcium ionophore A23187, equine peripheral blood neutrophils rapidly released extracellular DNA in a dose-dependent manner (Figure 1A). Extracellular DNA was observed as early as 1 h post-treatment, consistent with previous literature using A23187. Using electron microscopy, extruded strands of material were observed extending from stimulated cells (Figure 1A). To verify that the extracellular DNA was due to NET formation, immunofluorescence and immunogold labeling was performed on stimulated neutrophils at the conclusion of the assay (Figure 1B). NETs were identified via co-localization of DNA and citrullination of histone 3, a marker of NET formation. Long strands of CitH3 positive DNA were frequently observed within stimulated wells but were rarely present in non-stimulated wells (Figure 1B). Myeloperoxidase was observed adherent to the strands of extruded DNA, another marker of NET formation. To confirm that A23187 stimulated equine NET release in a NOX-independent manner, neutrophils were pretreated with NADPH-oxidase inhibitor DPI prior to stimulation. No alteration in extracellular DNA kinetics was observed, consistent with A23187 producing NETs via a NOX-independent mechanism (Figure 1C). This is consistent with ex vivo NET formation in response to A23187 in DPI-treated equine neutrophils (33). As expected, DPI was able to robustly inhibit A23187-stimulated respiratory burst (Figure 1D).

### 3.2 Equine neutrophils weakly produce NETs in an NOX-dependent fashion in response to PMA stimulation

**Figure 2:**
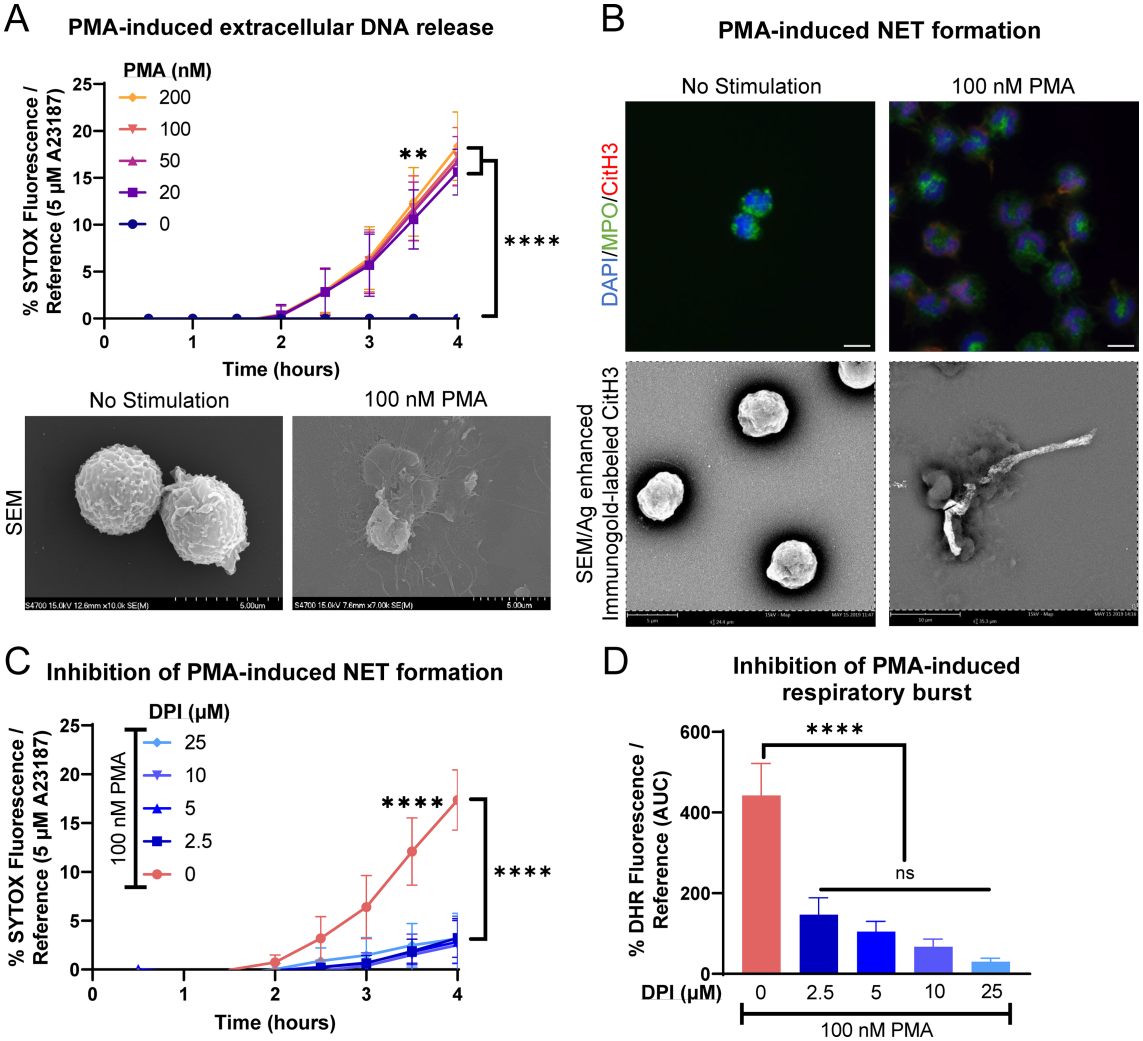
Equine neutrophils exhibit modest NOX-dependent NET formation in response to chemical stimulus with PMA. **(A)** Measurement of extracellular DNA released over time (0–4 h) from equine neutrophils stimulated in vitro with increasing concentrations of PMA (10–200 nM). Extracellular DNA was measured with SYTOX fluorescence. Due to high individual variation, all neutrophil responses were standardized to each animal’s response to 5 μM A23187. *n* = 3 horses. Significance indicated for all concentrations of PMA at indicated timepoints: ** *p* < 0.01; **** *p* < 0.0001. SEM images demonstrate extracellular contents extruded from neutrophils stimulated with 100 nM PMA. Scale bar 5 μm. **(B)** Confirmation of NET formation induced 4 h post-PMA stimulation in equine neutrophils. Myeloperoxidase (MPO) in green marks neutrophils and citrullinated H3 (CitH3) in purple identifies citrullinated histones, a marker of NET formation. Nuclei were stained with DAPI. SEM images demonstrate silver-enhanced immunogold labeled citrullinated H3 (CitH3) associated with some of the extruded contents. Scale bar 10 μm. **(C)** Measurement of NET formation over time (0–4 h) from equine neutrophils stimulated with PMA and incubated with increasing concentrations of NADPH-oxidase inhibitor DPI. Neutrophils were incubated with DPI prior to exposure to PMA. *n* = 3 horses. **** *p* < 0.0001. **(D)** Area under the curve (0–4 h) of respiratory burst from equine neutrophils stimulated with PMA and incubated with increasing concentrations of DPI. Neutrophils were incubated with DPI prior to exposure to PMA. *n* = 3 horses. **** *p* < 0.0001.

Equine peripheral blood neutrophils produced modest amounts of extracellular DNA when stimulated with PMA, a classical agonist for respiratory burst and NET induction (Figure 2A). Neutrophils did not exhibit a dose-dependent extracellular DNA release in response to PMA stimulation. NET formation was examined using immunofluorescence and immunogold labeling for citrullinated histone 3. Neutrophils exhibited fewer long strands of DNA with CitH3 positivity, with the majority of CitH3 deposition present intracellularly (associated with nuclei) or as small fine strands (Figure 2B). The extrusion of extracellular DNA was significantly inhibited by the addition of DPI, suggesting that the modest NET formation produced was via a NOX-dependent pathway (Figure 2C). Significant inhibition of respiratory burst was also observed after pre-incubation with DPI (Figure 2D).

### 3.3 Equine neutrophils produce significantly more NETs in response to calcium ionophore stimulation as compared to PMA stimulation

**Figure 3:**
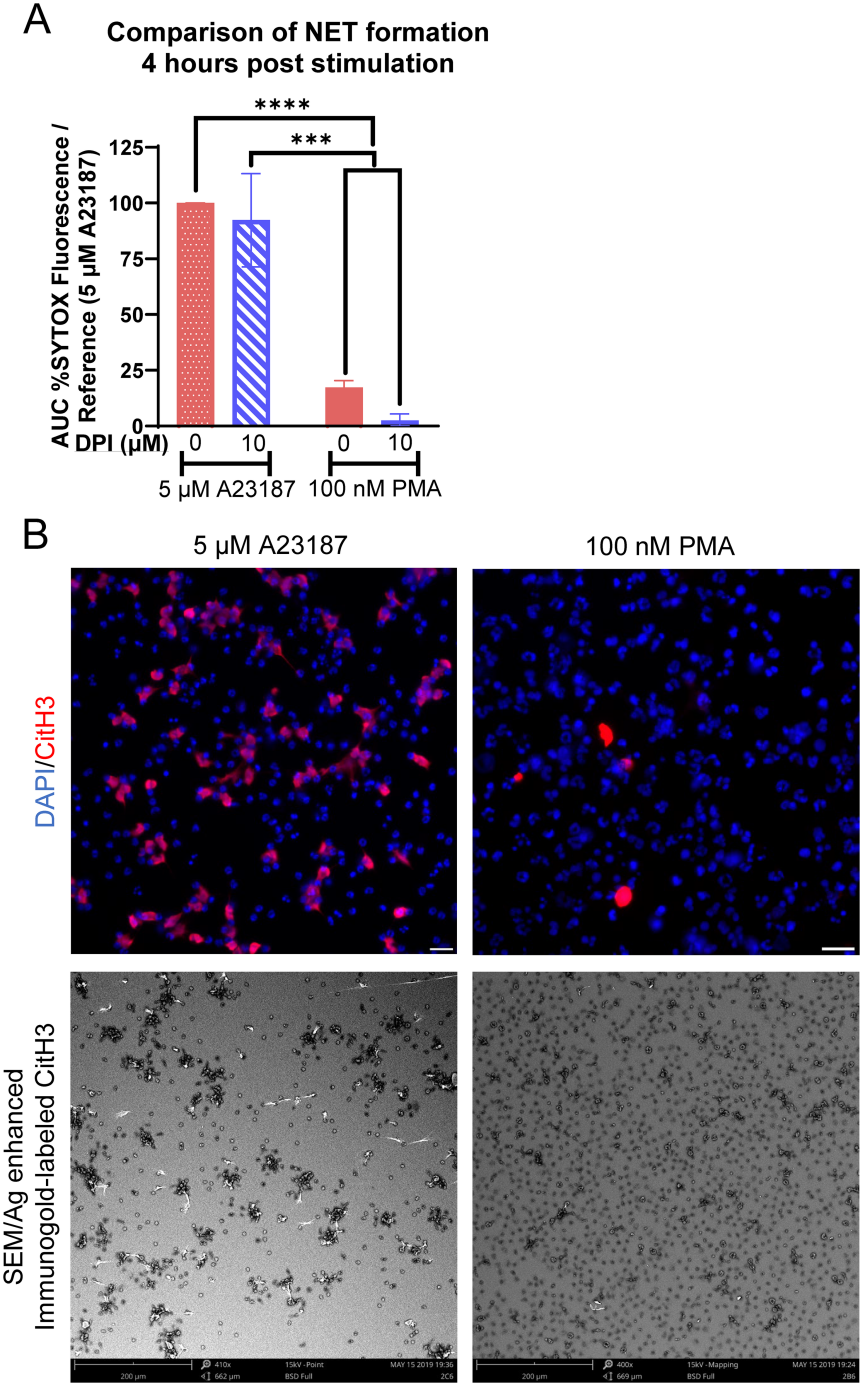
Kinetics of NOX-independent and NOX-dependent NET formation in equine neutrophils are distinct. **(A)** Area under the curve (AUC; 0–4 h) of respiratory burst from equine neutrophils stimulated with 5 μM A23187 or 100 nM PMA and incubated with 10 μM DPI. Neutrophils were incubated with DPI prior to exposure to PMA or A23187. Responses were normalized to the AUC of SYTOX fluorescence of neutrophils stimulated with 5 μM A23187. *n* = 3 horses. *** *p* < 0.001; **** *p* < 0.0001. **(B)** Comparison of NET formation induced 4 h post stimulation in equine neutrophils in both immunofluorescent images and SEM. Citrullinated H3 (CitH3) identifies citrullinated histones, a marker of NET formation. Scale bar 20 μm (immunofluorescence) or 200 μm (SEM).

We subsequently directly compared the relative amounts of NET formation utilizing the two agonists of NOX-dependent and NOX-independent NET formation. By 4 h post-stimulation, equine neutrophils exposed to A23187 had produced significantly more extracellular DNA as compared to PMA, and pre-incubation with DPI inhibited PMA-induced production of extracellular DNA (Figure 3A). This was validated with immunofluorescence and immunogold labeling for CitH3 (Figure 3B), where fewer neutrophils exhibited immunopositivity after PMA stimulation as compared to A23187. These results suggest that, in equine neutrophils, PMA is a weak agonist of NET formation as compared to a calcium ionophore.

### 3.4 Clinically relevant bacteria cause distinct equine neutrophil responses

**Figure 4:**
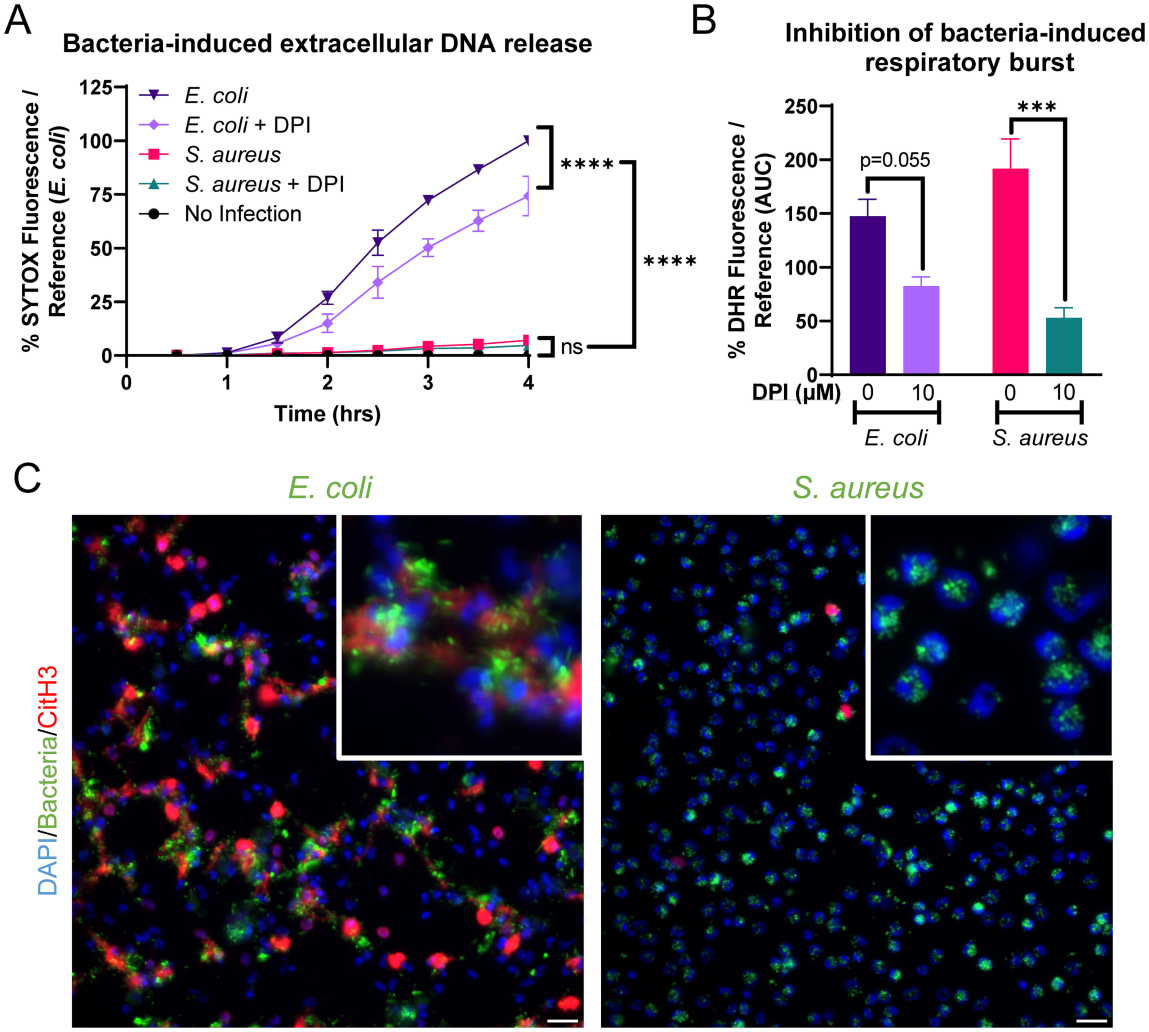
Equine NET formation is dependent on the bacterial stimulus. **(A)** Measurement of extracellular DNA released over time (0–4 h) from equine neutrophils stimulated in vitro with *E. coli* and *S. aureus* bacteria and inhibited with 10 uM DPI. Extracellular DNA was measured with SYTOX fluorescence. Due to high individual variation, all neutrophil responses were standardized to each animal’s response to *E. coli* stimulation (100%). *n* = 3 horses. Significance for the indicated groups at the indicated timepoints: *p < 0.05; ** *p* < 0.01; *** *p* < 0.001; **** *p* < 0.0001. **(B)** Area under the curve (0–4 h) of respiratory burst from equine neutrophils stimulated with bacteria and incubated with 10 uM DPI. Neutrophils were incubated with DPI prior to exposure to bacteria. *** *p* < 0.001. **(C)** Representative images of equine NET formation in response to stimulation with *E. coli* and *S. aureus*. Scale bar 10 μm.

Equine peripheral blood neutrophils released significantly higher amounts of extracellular DNA in response to *E. coli* (ATCC 25922) as compared to *S. aureus* (USA300) or no stimulation (Figure 4A). DPI modestly but incompletely inhibited extracellular DNA release in the face of *E. coli* stimulation, suggesting a minor contribution of a NOX-dependent pathway to NET formation in response to this bacterial agonist. DPI also inhibited the respiratory burst stimulated by the bacteria (Figure 4B). The difference in NET formation was identified via immunofluorescence for CitH3 (Figure 4C). After stimulation with *E. coli*, equine neutrophils extruded many strands of extracellular DNA with CitH3 deposition. Several of these strands appeared to be adherent to bacterial clumps (Figure 4C, *E. coli* inset). In contrast*, S. aureus* stimulation resulted in minimal NET formation, with bacteria primarily observed within neutrophils (Figure 4C, *S. aureus* inset), consistent with phagocytosis.

### 3.5 Inhibition of autophagy impairs NET extrusion from equine neutrophils

While an appropriate immunologic response is important for eradication of pathogens from the body, there is also evidence that excessive NET formation is detrimental to the host. We suspect this may also be the case in clinical cases of equine sepsis secondary to bacteria such as *E. coli* that induce robust NET formation ex vivo. Therefore, we wished to study a potential therapeutic that would limit NET formation from equine neutrophils. Autophagy has previously been identified as an important component of neutrophil functions in other species, including respiratory burst and NET formation.

**Figure 5:**
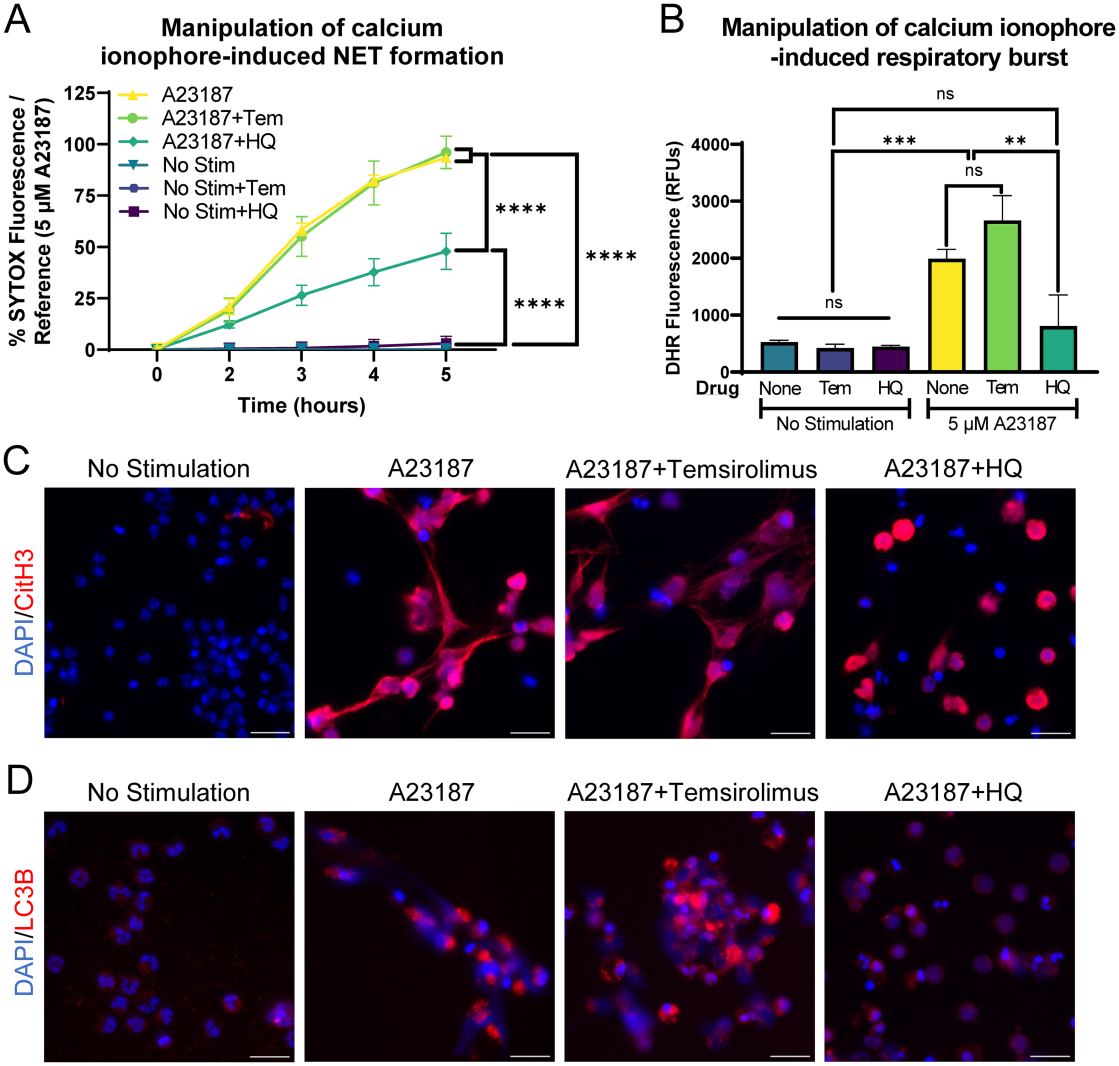
Autophagy inhibition limits calcium ionophore-induced NET release from equine neutrophils. **(A)** Measurement of extracellular DNA released over time (0–5 h) from equine neutrophils stimulated in vitro with 5 μM A23187 and incubated with either 100 nM temsirolimus (Tem) or 25 μM hydroxychloroquine (HQ). Extracellular DNA was measured with SYTOX fluorescence. Due to high individual variation, all neutrophil responses were standardized to each animal’s response to 5 μM A23187 (100%). *n* = 3 horses. Significance for the indicated groups at final timepoint:**** *p* < 0.0001. **(B)** Area under the curve (0–5 h) of respiratory burst from equine neutrophils stimulated with 5 μM A23187 and incubated with either 100 nM temsirolimus (Tem) or 25 μM hydroxychloroquine (HQ). *n* = 3 horses. ns = not significant; ** *p* < 0.01; *** *p* < 0.001. **(C)** Representative images of equine NET formation indicated by CitH3 immunofluorescence induced by 5 μM A23187 and response to 100 nM temsirolimus (Tem) and 25 μM hydroxychloroquine (HQ). Scale bar 20 μm. **(D)** Confirmation of autophagy modulation with LC3B immunofluorescence in the indicated conditions. Scale bar 20 μm.

Equine peripheral blood neutrophils were co-incubated with autophagy modulators and A23187, since A23187 was a robust inducer of NET formation in our model. Autophagy inhibition with hydroxychloroquine (HQ) significantly decreased A23187-induced extracellular DNA release, whereas autophagy induction with temsirolimus (TEM) did not alter the kinetics of extracellular DNA release (Figure 5A). Similarly, autophagy inhibition significantly impaired respiratory burst function (Figure 5B). NET induction was then examined via immunofluorescence for citrullinated histone 3. The inhibition of autophagy with hydroxychloroquine altered the morphology of CitH3 immunopositivity, where the majority of CitH3-positive DNA remained in a non-extruded state (Figure 5C). This suggests that the inhibition of autophagy effectively impaired the neutrophil’s ability to extrude DNA, but did not stop the deposition of citrulline on histone 3. The physiological consequence(s) of this altered morphology is unknown. Autophagy modulation was confirmed with immunofluorescence for LC3B, a marker of autophagy. A23187-stimulated neutrophils exhibited increased LC3B staining, and this was subjectively decreased in wells treated with autophagy inhibitor HQ (Figure 5D).

## 4 Discussion

We demonstrated here that *ex vivo* equine peripheral blood neutrophils produce robust NETs with associated citrullinated histone 3 and myeloperoxidase in response to specific agonists. Calcium ionophore and *E. coli* stimulated the most robust NET production in equine neutrophils, while PMA and *S. aureus* primarily stimulated respiratory burst, not NET production. We also verified that NOX-independent and NOX-dependent pathways of NET generation occur in equine neutrophils, similar to a recent publication (33). Finally, we determined that extrusion of extracellular DNA as part of the NET production process in equine neutrophils is reliant on intact autophagy machinery.

The use of fluorescent microscopy is key to characterize NET formation and differentiate it from DNA release of a different mechanism such as necrosis. NET formation is confirmed by the colocalization of NET associated proteins, such as CitH3 and myeloperoxidase with extracellular and sometimes intracellular DNA (2, 21, 33, 39–42). Citrullination of histones is a NET-specific marker performed by the peptidyl arginine deiminase (PAD) enzymes that post-translationally converts arginine, a positively charged residue, to citrulline, a neutral residue (43). NET formation in ex vivo equine neutrophils is dependent on intact PAD activity (33). It has previously been demonstrated that NET formation in equines can be labeled with an extracellular histone 2B marker (19) and histone H4 marker (33). We validated our measurement of extracellular DNA by immunofluorescent labeling with CitH3 and identification of myeloperoxidase granules adherent to strands of CitH3-positive DNA. These methods will be useful to other equine researchers particularly given the recent interest in cfDNA measurements in various body fluids such as bronchoalveolar lavages and peritoneal fluid.

PMA is a poor inducer of NET formation in equine neutrophils, in contrast to human neutrophils. PMA was used in the first publication alluding to NET formation (44), and since has been a popular stimulus in a wide variety of studies examining human diseases such as diabetes (12, 45), cystic fibrosis (46–48), rheumatoid arthritis (11, 49), chronic granulomatous disease (15, 50), and sepsis (39, 51). We did not observe any dose-dependent difference in equine neutrophils over a range of 20 nM-100 nM, although a previous equine study indicated that high doses (100 nM) are associated with apoptosis rather than NET production and that the ideal dose for equine NET formation was 25 nM. It is possible that differences in assay methods, neutrophil origin, or evaluation of NET formation may have caused the differences in conclusion between the two studies. Additionally, that study did not utilize A23187 so the relative NET production to different stimuli is difficult to directly compare. The phenotypic difference in our neutrophilic response to PMA vs. A23187 is particularly apparent in the scanning electron microscopy images, where the calcium ionophore-stimulated neutrophil exhibits the classic stranding of NET formation with small granules adherent to the strand. These images are consistent with those of Quiroga et al., where A23187 induced long filamentous extrusion compared to PMA (33).

Given that equine neutrophils modestly differ from other species with regards to NET formation to classical agonists, we were curious if these differences also extend to clinically relevant bacteria. In humans, gram-positive pathogens such as *S. aureus* are strong inducers of rapid NET formation via a NOX-independent mechanism downstream of TLR2 activation (52, 53). One theory is that human neutrophils produce NETs to immobilize these bacteria since *S. aureus* has defensive mechanisms against oxidant damage by neutrophils and thus survive classic neutrophil attacks such as phagocytosis. In contrast to human neutrophils, equine neutrophils primarily phagocytose *S. aureus* with minimal NET production. However, we did not study whether the phagocytic ability of equine neutrophils successfully killed the bacteria, which might have provided a potential explanation as to why equine NET formation is poorly stimulated by *S. aureus*.

In contrast to *S. aureus*, we found that *E. coli* stimulation is a robust driver of NET formation in equine neutrophils. Gram negative pathogens, such as *E. coli* and *Pseudomonas aeruginosa*, have been reported to drive NET formation in human neutrophils via a NOX-dependent mechanism downstream of TLR4 activation (42, 50). We found a minor but statistically significant contribution of the NOX pathway to NET production in *E. coli* stimulated equine neutrophils. It is well known that equines have a high sensitivity to *E. coli* derived LPS, resulting in tumor necrosis factor (TNF) and other cytokine production (35). Whether this plays a role in the NET formation observed in response to *E. coli* stimulation ex vivo is unknown. A recent equine study using *E. coli* derived LPS (no bacteria) did not identify robust stimulation of NET release (33). It is unknown whether this disparate result is due to the serotype evaluated or if there are other components included with live bacteria that are sensed by the equine neutrophils in our study which subsequently induce NET release.

We demonstrated that, similar to other species, equine NET formation is reliant on autophagy machinery. Hydroxychloroquine has recently been demonstrated to inhibit PAD4 function in addition to autophagy (54), thus there may be multiple simultaneous mechanisms at play. However, the changes observed in LC3B immunofluorescence are suggestive of autophagy’s direct role in equine NET formation. Consistent with our findings in equine neutrophils, autophagy inhibition in human neutrophils causes a similar phenotype of non-extruded CitH3 positivity (23). Survival of sepsis and ischemia/reperfusion injury in mice has been enhanced after treatment to block NET formation with inhibitors of PAD and autophagy (55). This supports autophagy inhibition as a potential therapeutic to block excessive NET release in equine diseases.

The large numbers of NETs that are produced by equine neutrophils in response to calcium ionophore and *E. coli* are concerning for potential systemic or local complications secondary to NET formation. The DNA and NET-associated proteins released into the extracellular space cause local inflammation (56), and when the NETs get into the bloodstream they can cause thrombosis and vasculitis (7, 10, 57). Increased biomarkers of NETs are associated with respiratory failure in COVID19-positive patients, suggesting that NET formation also contributes to respiratory dysfunction (58). NETs have been found in bronchoalveolar lavage fluid from horses with severe equine asthma, and the degree of NET burden correlates to the disease severity (31). While glucocorticosteroids decreased respiratory NET formation in equine asthma (28), these drugs have potentially severe side effects such as laminitis (59). Therefore, alternative therapies that target NET release should be identified.

The objective of this study was to characterize equine NET formation ex vivo. We demonstrated that horses have differing degrees of NET formation in response to chemical and bacterial stimuli, but that the underlying processes controlling NET formation in horses is consistent with that published for other species. This study was limited by the exclusive use of small numbers of peripheral blood neutrophils studied ex vivo obtained from healthy horses. Future studies should examine the influence of systemic disease and *in vivo* environment on equine NET formation in response to different agonists, including different serotypes of *E. coli* and other relevant equine bacteria. We were unable to perform SEM on bacterial samples due to institutional restrictions on machine usage, although this would have allowed further characterization of the bacterial response. Autophagy modulation was performed using our reference agonist, A23187, to determine if equine neutrophils responded similarly to human neutrophils. Future studies could expand the agonists used to include *E. coli* as a biologically relevant stimulus, and test different autophagy inhibitors to identify a potential therapeutic for excessive NET release in horses. As NET formation appears to be a relevant component of diseases such as asthma that can severely impact equine health, interventions aimed at modifying NET production could be a major milestone in treatment.

## Data Availability

The raw data supporting the conclusions of this article will be made available by the authors, without undue reservation.
